# Supplementary material for: Measuring socioeconomic inequalities in prenatal HIV test service uptake for prevention of mother to child transmission of HIV in East Africa: A decomposition analysis
Source: PLoS One. 2022 Aug 23;17(8):e0273475. doi: 10.1371/journal.pone.0273475 (PMC9398021; doi:10.1371/journal.pone.0273475)

Supplementary 1: Erreygers’ normalized concentration indexes and curves for prenatal HIV test uptake across East African countries

| Countries | Weighted Sample | Concentration Index | Std. Error | P-value |
| --- | --- | --- | --- | --- |
| Burundi | 5,412 | 0.0506 | 0.0096 | < 0.001 |
| Comoros | 1,298 | 0.1811 | 0.0239 | < 0.001 |
| Ethiopia | 4308 | 0.4033 | 0.0159 | < 0.001 |
| Kenya | 7,357 | 0.1324 | 0.0064 | < 0.001 |
| Malawi | 6,693 | 0.0346 | 0.0087 | < 0.001 |
| Mozambique | 4,913 | 0.4030 | 0.0155 | < 0.001 |
| Rwanda | 3,236 | 0.0187 | 0.0059 | < 0.01 |
| Uganda | 5,901 | 0.0739 | 0.0082 | < 0.001 |
| Zambia | 3,905 | 0.0951 | 0.0091 | < 0.001 |
| Zimbabwe | 2,454 | 0.0994 | 0.0138 | < 0.001 |
| East Africa | 45,476 | 0.1594 | 0.0042 | < 0.001 |


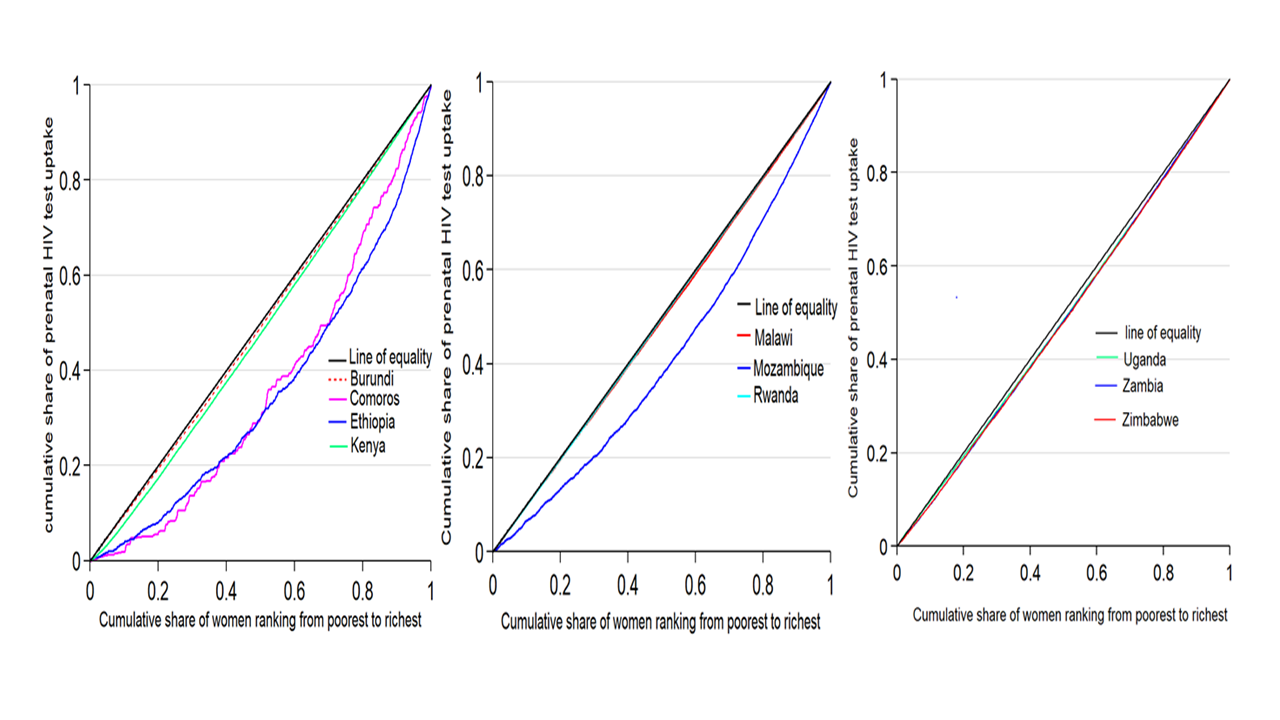

Supplement: S1 File — (DOCX) [file pone.0273475.s001.docx]
