# Supplementary material for: Measuring socioeconomic inequalities in prenatal HIV test service uptake for prevention of mother to child transmission of HIV in East Africa: A decomposition analysis
Source: PLoS One. 2022 Aug 23;17(8):e0273475. doi: 10.1371/journal.pone.0273475 (PMC9398021; doi:10.1371/journal.pone.0273475)
Supplement: S2 File — (DOCX) [file pone.0273475.s002.docx]

Supplementary table 2a: A decomposition analysis of socioeconomic inequalities in prenatal HIV testing for PMTCT among mother aged 15-49 years in Burundi and Comoros

|  | **Burundi** | | | | | | **Comoros** | | | | | |
| --- | --- | --- | --- | --- | --- | --- | --- | --- | --- | --- | --- | --- |
|  |  |  |  | **Contribution** | | |  |  |  | **Contribution** | | |
| **Characteristics** | **Marginal effect** | **Elasticity** | **ECI** | **Value** | **Percentage** | **Summed %** | **Coefficient** | **Elasticity** | **ECI** | **Value** | **Percentage** | **Summed %** |
| Residence(base=Rural) |  |  |  |  |  |  |  |  |  |  |  |  |
| Urban | 0.0807** | 0.0291 | 0.2533 | 0.0073 | 14.59 | 14.59 | 0.0560* | 0.0636 | 0.2471 | 0.0157 | 8.67 | 8.67 |
| Maternal age (base=15-19 years) |  |  |  |  |  |  |  |  |  |  |  |  |
| 20-34 years | 0.0026 | 0.0054 | 0.0254 | 0.0001 | 0.27 | -0.25 | -0.0067 | -0.0129 | 0.1678 | -0.0021 | -1.19 | -0.76 |
| 35-49 years | 0.0123 | -0.0114 | 0.0232 | -0.0002 | -0.52 |  | -0.0258 | -0.0215 | -0.0363 | 0.0007 | 0.43 |  |
| Maternal education (base= No education) |  |  |  |  |  |  |  |  |  |  |  |  |
| Primary | 0.0025 | 0.0044 | 0.0536 | 0.0002 | 0.47 | 0.01 | -0.0214 | -0.0214 | 0.0249 | -0.0005 | -0.29 | 3.26 |
| Secondary and higher | -0.0018 | -0.0008 | 0.2632 | -0.0002 | -0.46 |  | 0.0126 | 0.0161 | 0.3985 | 0.0064 | 3.55 |  |
| Maternal occupation (base= Not working) |  |  |  |  |  |  |  |  |  |  |  |  |
| Professional work | -0.0205 | -0.0074 | 0.2189 | -0.0016 | -3.23 | 32.91 | -0.0485 | -0.0314 | 0.1655 | -0.0052 | -2.87 | -0.39 |
| Nonprofessional work | -0.0173 | -0.0581 | -0.3147 | 0.0182 | 36.14 |  | -0.0557 | -0.0416 | -0.1078 | 0.0044 | 2.48 |  |
| Partner education (base= No education) |  |  |  |  |  |  |  |  |  |  |  |  |
| Primary | -0.0112 | -0.0231 | 0.0845 | -0.0019 | -3.86 | -6.47 | 0.0596* | 0.0626 | -0.0496 | -0.0031 | -1.71 | 29.8 |
| Secondary and Higher | -0.0099 | -0.0048 | 0.2699 | -0.0013 | -2.61 |  | 0.1039*** | 0.1565 | 0.3647 | 0.0570 | 31.51 |  |
| Sex of the head of household (base=Male) |  |  |  |  |  |  |  |  |  |  |  |  |
| Female | 0.0139 | 0.0100 | 0.0007 | 0.0000 | 0.00 | 0.00 | -0.0332 | -0.0422 | 0.0043 | -0.0001 | -0.10 | -0.10 |
| Household family size(base=1-3) |  |  |  |  |  |  |  |  |  |  |  |  |
| 4-6 | 0.0057 | 0.0117 | -0.0915 | -0.0010 | -2.12 | -2.12 | -0.0158 | -0.0284 | -0.0219 | 0.0006 | 0.34 | 2.87 |
| >=7 | 0.0001 | 0.0002 | 0.1686 | 0.0000 | 0.00 |  | 0.0499 | 0.0849 | 0.0541 | 0.0045 | 2.53 |  |
| Household wealth index (base=Poorest) |  |  |  |  |  |  |  |  |  |  |  |  |
| Poorer | 0.0067 | 0.0057 | -0.3053 | -0.0017 | -3.47 | 16.64 | 0.1033* | 0.0834 | -0.3638 | -0.0303 | -16.76 | 32.51 |
| Middle | 0.0147 | 0.0125 | 0.0584 | 0.0007 | 1.44 |  | 0.1190** | 0.0964 | -0.0374 | -0.0036 | -1.99 |  |
| Wealthier | 0.0050 | 0.0041 | 0.3965 | 0.0016 | 3.21 |  | 0.1242** | 0.1028 | 0.3008 | 0.0309 | 17.07 |  |
| Wealthiest | 0.0240 | 0.0149 | 0.5244 | 0.0078 | 15.46 |  | 0.1068* | 0.0918 | 0.6747 | 0.0619 | 34.19 |  |
| Read newspaper (base= No) |  |  |  |  |  |  |  |  |  |  |  |  |
| Yes | -0.0111 | -0.0019 | 0.0670 | -0.0001 | -0.25 | -0.25 | 0.1035*** | 0.0619 | 0.2308 | 0.0143 | 7.89 | 7.89 |
| Listened to the radio (base= No) |  |  |  |  |  |  |  |  |  |  |  |  |
| Yes | 0.0501*** | 0.0877 | 0.3930 | 0.0345 | 68.16 | 68.16 | -0.0164 | -0.0330 | 0.4098 | -0.0135 | -7.48 | -7.48 |
| Watched television (base= No) |  |  |  |  |  |  |  |  |  |  |  |  |
| Yes | -0.0048 | -0.0013 | 0.1589 | -0.0002 | -0.43 | -0.43 | -0.0054 | -0.0145 | 0.4368 | -0.0063 | -3.49 | -3.49 |
| Explained |  |  |  |  |  | 122.79 |  |  |  |  |  | 72.78 |
| Residual |  |  |  |  |  | -22.79 |  |  |  |  |  | 27.22 |
| Total |  |  |  |  |  | 100 |  |  |  |  |  | 100 |

|  | **Ethiopia** | | | | | | **Kenya** | | | | | |
| --- | --- | --- | --- | --- | --- | --- | --- | --- | --- | --- | --- | --- |
|  |  |  |  | **Contribution** | | |  |  |  | **Contribution** | | |
| **Characteristics** | **Marginal effect** | **Elasticity** | **ECI** | **Value** | **Percentage** | **Summed %** | **Coefficient** | **Elasticity** | **ECI** | **Value** | **Percentage** | **Summed %** |
| Residence(base=Rural) |  |  |  |  |  |  |  |  |  |  |  |  |
| Urban | 0.1825*** | 0.0881 | 0.3563 | 0.0313 | 8.21 | 7.78 | -0.0119 | -0.0170 | 0.6225 | -0.0106 | -8.02 | -8.46 |
| Maternal age (base=15-19 years) |  |  |  |  |  |  |  |  |  |  |  |  |
| 20-34 years | 0.0327 | 0.0666 | -0.0849 | 0.0056 | 1.40 | 1.2 | -0.0036 | -0.0068 | 0.0631 | -0.0004 | -0.32 | -0.32 |
| 35-49 years | 0.0655** | 0.0520 | -0.0157 | -0.0008 | -0.20 |  | 0.0004 | 0.0002 | -0.0554 | -0.0000 | 0.00 |  |
| Maternal education (base= No education) |  |  |  |  |  |  |  |  |  |  |  |  |
| Primary | 0.1215*** | 0.1243 | 0.1513 | 0.0188 | 4.66 | 8.34 | 0.0362** | 0.0792 | -0.2013 | -0.0159 | -12.04 | 26.74 |
| Secondary and higher | 0.1888*** | 0.0672 | 0.2209 | 0.0148 | 3.68 |  | 0.0781*** | 0.1062 | 0.4833 | 0.0513 | 38.78 |  |
| Maternal occupation (base= Not working) |  |  |  |  |  |  |  |  |  |  |  |  |
| Professional work | 0.0045 | 0.0029 | 0.1778 | 0.0005 | 0.13 | -0.74 | -0.0021 | -0.0013 | 0.1989 | -0.0002 | -0.20 | 1.88 |
| Nonprofessional work | 0.0440** | 0.0435 | -0.0810 | -0.0035 | -0.87 |  | -0.0115 | -0.0140 | -0.1961 | 0.0027 | 2.08 |  |
| Partner education (base= No education) |  |  |  |  |  |  |  |  |  |  |  |  |
| Primary | -0.0210 | -0.0341 | 0.0780 | -0.0026 | -0.66 | 1.47 | 0.0275* | 0.0556 | -0.2412 | -0.0134 | -10.14 | 14.08 |
| Secondary and Higher | 0.0532* | 0.0307 | 0.2802 | 0.0086 | 2.13 |  | 0.0402* | 0.0642 | 0.4992 | 0.0320 | 24.22 |  |
| Sex of the head of household (base=Male) |  |  |  |  |  |  |  |  |  |  |  |  |
| Female | -0.0537* | -0.0291 | 0.0144 | -0.0004 | -0.10 | -0.10 | -0.0038 | -0.0040 | -0.0869 | 0.0003 | 0.26 | 0.26 |
| Household family size(base=1-3) |  |  |  |  |  |  |  |  |  |  |  |  |
| 4-6 | -0.0330 | -0.0673 | 0.0490 | -0.0033 | -0.81 | 1.71 | 0.0127 | 0.0260 | 0.0448 | 0.0011 | 0.88 | 3.32 |
| >=7 | -0.1063*** | -0.1555 | -0.0653 | 0.0101 | 2.52 |  | -0.0136 | -0.0166 | -0.1942 | 0.0032 | 2.44 |  |
| Household wealth index (base=Poorest) |  |  |  |  |  |  |  |  |  |  |  |  |
| Poorer | 0.0883** | 0.0727 | -0.5183 | -0.0377 | -9.35 | 38.81 | 0.0293* | 0.0215 | -0.4369 | -0.0094 | -7.10 | 57.94 |
| Middle | 0.1559*** | 0.1773 | -0.1579 | -0.0280 | -6.94 |  | 0.0381* | 0.0306 | -0.1700 | -0.0052 | -3.93 |  |
| Wealthier | 0.2355*** | 0.2801 | 0.5269 | 0.1476 | 36.60 |  | 0.0459** | 0.0403 | 0.1836 | 0.0074 | 5.59 |  |
| Wealthiest | 0.3181*** | 0.1651 | 0.4518 | 0.0746 | 18.50 |  | 0.1058*** | 0.1209 | 0.8162 | 0.0987 | 74.56 |  |
| Read newspaper (base= No) |  |  |  |  |  |  |  |  |  |  |  |  |
| Yes | 0.0455 | 0.0128 | 0.1416 | 0.0018 | 0.45 | 0.45 | 0.0346* | 0.0403 | 0.3105 | 0.0125 | 9.45 | 9.45 |
| Listened to the radio (base= No) |  |  |  |  |  |  |  |  |  |  |  |  |
| Yes | 0.0312 | 0.0345 | 0.3149 | 0.0108 | 2.69 | 2.69 | 0.0151 | 0.0476 | 0.3096 | 0.0147 | 11.14 | 11.14 |
| Watched television (base= No) |  |  |  |  |  |  |  |  |  |  |  |  |
| Yes | 0.0259 | 0.0195 | 0.3777 | 0.0074 | 1.83 | 1.83 | 0.0160 | 0.0270 | 0.6681 | 0.0180 | 13.65 | 13.65 |
| Explained |  |  |  |  |  | 63.44 |  |  |  |  |  | 129.68 |
| Residual |  |  |  |  |  | 36.56 |  |  |  |  |  | -29.68 |
| Total |  |  |  |  |  | 100 |  |  |  |  |  | 100 |

Supplementary table 2b: A decomposition analysis of socioeconomic inequalities in prenatal HIV testing for PMTCT among mother aged 15-49 years in Ethiopia and Kenya

|  | **Malawi** | | | | | | **Mozambique** | | | | | |
| --- | --- | --- | --- | --- | --- | --- | --- | --- | --- | --- | --- | --- |
| **Characteristics** | **Marginal effect** | **Elasticity** | **ECI** | **Contribution** | | | **Marginal effect** | **Elasticity** | **ECI** | **Contribution** | | |
|  |  |  |  | **Value** | **Percentage** | **Summed %** |  |  |  | **Value** | **Percentage** | **Summed %** |
| Residence(base=Rural) |  |  |  |  |  |  |  |  |  |  |  |  |
| Urban | 0.0130 | 0.0070 | 0.3669 | 0.0026 | 7.52 | 7.52 | 0.0131 | 0.0144 | 0.5129 | 0.0074 | 1.84 | 1.84 |
| Maternal age (base=15-19 years) |  |  |  |  |  |  |  |  |  |  |  |  |
| 20-34 years | 0.0082 | 0.0126 | 0.0840 | 0.0010 | 3.08 | 3.35 | 0.0635*** | 0.1038 | -0.0069 | -0.0007 | -0.17 | -0.17 |
| 35-49 years | -0.0146 | -0.0089 | -0.0105 | 0.0001 | 0.27 |  | 0.0013 | 0.009 | -0.0836 | -0.0000 | 0.00 |  |
| Maternal education (base= No education) |  |  |  |  |  |  |  |  |  |  |  |  |
| Primary | 0.0161 | 0.0432 | -0.2267 | -0.0098 | -28.34 | 2.55 | 0.0560*** | 0.1161 | 0.0089 | 0.0010 | 0.25 | 9.49 |
| Secondary and higher | 0.0335* | 0.0284 | 0.3760 | 0.0106 | 30.89 |  | 0.2310*** | 0.1166 | 0.3197 | 0.0372 | 9.24 |  |
| Maternal occupation (base= Not working) |  |  |  |  |  |  |  |  |  |  |  |  |
| Professional work | 0.0334 | 0.0123 | 0.1716 | 0.0021 | 6.11 | -43.48 | 0.0555 | 0.0235 | 0.1485 | 0.0034 | 0.86 | 14.24 |
| Nonprofessional work | 0.0279** | 0.0644 | -0.2664 | -0.0171 | -49.59 |  | -0.1262*** | -0.1726 | -0.3126 | 0.0539 | 13.38 |  |
| Partner education (base= No education) |  |  |  |  |  |  |  |  |  |  |  |  |
| Primary | 0.0520*** | 0.1138 | -0.3580 | -0.0407 | -117.78 | 4.54 | 0.0530** | 0.1162 | -0.1793 | -0.0208 | -5.17 | 4.78 |
| Secondary and Higher | 0.0640*** | 0.0897 | 0.4714 | 0.0423 | 122.32 |  | 0.0918** | 0.0714 | 0.3924 | 0.0280 | 9.95 |  |
| Sex of the head of household(base=Male) |  |  |  |  |  |  |  |  |  |  |  |  |
| Female | 0.0048 | 0.0046 | -0.1386 | -0.0006 | -1.84 | -1.84 | 0.0396* | 0.0464 | 0.0877 | 0.0040 | 1.01 | 1.01 |
| Household family size(base=1-3) |  |  |  |  |  |  |  |  |  |  |  |  |
| 4-6 | 0.0477*** | 0.1009 | -0.0071 | -0.0007 | -2.09 | 12.08 | 0.0125 | 0.0257 | -0.0745 | -0.0019 | -0.47 | 2.51 |
| >=7 | 0.0476*** | 0.0520 | 0.0942 | 0.0049 | 14.17 |  | 0.0648** | 0.0866 | 0.1390 | 0.0120 | 2.98 |  |
| Household wealth index (base=Poorest) |  |  |  |  |  |  |  |  |  |  |  |  |
| Poorer | -0.0156 | -0.0128 | -0.2961 | 0.0038 | 11.00 | 14.52 | 0.0778*** | 0.0705 | -0.2330 | -0.0164 | -4.07 | 33.05 |
| Middle | -0.0211 | -0.0172 | 0.0409 | -0.0007 | -2.04 |  | 0.1309*** | 0.1056 | 0.1379 | 0.0145 | 3.61 |  |
| Wealthier | -0.0092 | -0.0070 | 0.3332 | -0.0023 | -6.76 |  | 0.2280*** | 0.1604 | 0.3859 | 0.0619 | 15.35 |  |
| Wealthiest | 0.0093 | 0.0070 | 0.6015 | 0.0042 | 12.32 |  | 0.2797*** | 0.1541 | 0.4751 | 0.0732 | 18.16 |  |
| Read newspaper (base= No) |  |  |  |  |  |  |  |  |  |  |  |  |
| Yes | 0.0172 | 0.0112 | 0.1754 | 0.0019 | 5.71 | 5.71 | 0.0213 | 0.0087 | 0.2354 | 0.0020 | 0.50 | 0.50 |
| Listened to the radio (base= No) |  |  |  |  |  |  |  |  |  |  |  |  |
| Yes | 0.0301** | 0.0563 | 0.3367 | 0.0189 | 54.83 | 54.83 | -0.0004 | -0.0011 | 0.0834 | 0.0000 | 0.00 | 0.00 |
| Watched television (base= No) |  |  |  |  |  |  |  |  |  |  |  |  |
| Yes | -0.0141 | -0.0087 | 0.2952 | -0.0025 | -7.45 | -7.45 | 0.0405 | 0.0436 | 0.5315 | 0.0231 | 5.75 | 5.75 |
| Explained |  |  |  |  |  | 52.33 |  |  |  |  |  | 73 |
| Residual |  |  |  |  |  | 47.67 |  |  |  |  |  | 27 |
| Total |  |  |  |  |  | 100 |  |  |  |  |  | 100 |

Supplementary table 2c: A decomposition analysis of socioeconomic inequalities in prenatal HIV testing for PMTCT among mother aged 15-49 years in Malawi and Mozambique

Supplementary table 2d: A decomposition analysis of socioeconomic inequalities in prenatal HIV testing for PMTCT among mother aged 15-49 years in Rwanda and Uganda

|  | **Rwanda** | | | | | | **Uganda** | | | | | |
| --- | --- | --- | --- | --- | --- | --- | --- | --- | --- | --- | --- | --- |
| **Characteristics** | **Marginal effect** | **Elasticity** | **ECI** | **Contribution** | | | **Marginal effect** | **Elasticity** | **ECI** | **Contribution** | | |
|  |  |  |  | **Value** | **Percentage** | **Summed %** |  |  |  | **Value** | **Percentage** | **Summed %** |
| Residence(base=Rural) |  |  |  |  |  |  |  |  |  |  |  |  |
| Urban | 0.0009 | 0.0006 | 0.4194 | 0.0002 | 1.48 | 1.48 | 0.0344* | 0.0293 | 0.4044 | 0.0118 | 16.05 | 16.05 |
| Maternal age (base=15-19 years) |  |  |  |  |  |  |  |  |  |  |  |  |
| 20-34 years | 0.0018 | 0.0038 | 0.0321 | 0.0001 | 0.66 | 0.66 | 0.0224* | 0.0377 | 0.0442 | 0.0016 | 2.25 | 11.41 |
| 35-49 years | -0.0062 | -0.0054 | 0.0136 | 0.0000 | 0.00 |  | -0.0079 | -0.0049 | -0.0205 | 0.0001 | 13.66 |  |
| Maternal education (base= No education) |  |  |  |  |  |  |  |  |  |  |  |  |
| Primary | 0.0182** | 0.0523 | -0.1488 | -0.0078 | -41.65 | -28.57 | 0.0081 | 0.0197 | -0.3205 | -0.0063 | -8.57 | 35.42 |
| Secondary and higher | 0.0142 | 0.0084 | 0.2886 | 0.0024 | 13.08 |  | 0.0580** | 0.0691 | 0.4703 | 0.0325 | 43.99 |  |
| Maternal occupation (base= Not working) |  |  |  |  |  |  |  |  |  |  |  |  |
| Professional work | -0.0206 | -0.0110 | 0.2678 | -0.0029 | -15.86 | 38.92 | 0.0387* | 0.0324 | 0.3014 | 0.0097 | 13.21 | -3.6 |
| Nonprofessional work | -0.0083 | -0.0260 | -0.3941 | 0.0102 | 54.78 |  | 0.0118 | 0.0277 | -0.4474 | -0.0124 | -16.81 |  |
| Partner education (base= No education) |  |  |  |  |  |  |  |  |  |  |  |  |
| Primary | -0.0036 | -0.0104 | -0.0799 | 0.0008 | 4.46 | 16.33 | 0.0092 | 0.0199 | -0.2816 | -0.0056 | -7.59 | 19.34 |
| Secondary and Higher | 0.0171 | 0.0089 | 0.2495 | 0.0022 | 11.87 |  | 0.0323* | 0.0506 | 0.3929 | 0.0199 | 26.93 |  |
| Sex of the head of household(base=Male) |  |  |  |  |  |  |  |  |  |  |  |  |
| Female | -0.0084 | -0.0070 | -0.1242 | 0.0008 | 4.66 | 4.66 | 0.0233* | 0.0226 | 0.0132 | 0.0003 | 0.40 | 0.40 |
| Household family size(base=1-3) |  |  |  |  |  |  |  |  |  |  |  |  |
| 4-6 | 0.0071 | 0.0163 | -0.0286 | -0.0004 | -2.50 | -0.53 | 0.0333** | 0.0616 | -0.0205 | -0.0012 | -1.71 | -1.71 |
| >=7 | 0.0037 | 0.0033 | 0.1091 | 0.0003 | 1.97 |  | -0.0012 | -0.0018 | -0.0502 | 0.0000 | 0.00 |  |
| Household wealth index (base=Poorest) |  |  |  |  |  |  |  |  |  |  |  |  |
| Poorer | 0.0083 | 0.0069 | -0.3261 | -0.0022 | -12.06 | 69.37 | -0.0169 | -0.0126 | -0.3626 | 0.0045 | 6.21 | 46.2 |
| Middle | 0.0114 | 0.0096 | 0.0183 | 0.0001 | 0.94 |  | 0.0053 | 0.0043 | -0.0791 | -0.0003 | -0.46 |  |
| Wealthier | 0.0173 | 0.0141 | 0.3574 | 0.0050 | 27.10 |  | 0.0105 | 0.0088 | 0.2622 | 0.0023 | 3.13 |  |
| Wealthiest | 0.0236 | 0.0169 | 0.5886 | 0.0099 | 53.39 |  | 0.0397* | 0.0379 | 0.7272 | 0.0276 | 37.32 |  |
| Read newspaper (base= No) |  |  |  |  |  |  |  |  |  |  |  |  |
| Yes | 0.0107 | 0.0091 | 0.1966 | 0.0017 | 9.59 | 9.59 | 0.0031 | 0.0024 | 0.3208 | 0.0007 | 1.05 | 1.05 |
| Listened to the radio (base= No) |  |  |  |  |  |  |  |  |  |  |  |  |
| Yes | 0.0028 | 0.0091 | 0.2657 | 0.0024 | 13.00 | 13.00 | 0.0275** | 0.0798 | 0.2736 | 0.0218 | 39.55 | 39.55 |
| Watched television (base= No) |  |  |  |  |  |  |  |  |  |  |  |  |
| Yes | -0.0002 | -0.0004 | 0.3519 | -0.0001 | -0.83 | -0.83 | -0.0035 | -0.0039 | 0.4827 | -0.0019 | -2.56 | -2.56 |
| Explained |  |  |  |  |  | 124.08 |  |  |  |  |  | 162 |
| Residual |  |  |  |  |  | -24.08 |  |  |  |  |  | -62 |
| Total |  |  |  |  |  | 100 |  |  |  |  |  | 100 |

Supplementary table 2e: A decomposition analysis of socioeconomic inequalities in prenatal HIV testing for PMTCT among mother aged 15-49 years in Zambia and Zimbabwe

|  | **Zambia** | | | | | | **Zimbabwe** | | | | | |
| --- | --- | --- | --- | --- | --- | --- | --- | --- | --- | --- | --- | --- |
| **Characteristics** | **Marginal effect** | **Elasticity** | **ECI** | **Contribution** | | | **Marginal effect** | **Elasticity** | **ECI** | **Contribution** | | |
|  |  |  |  | **Value** | **Percentage** | **Summed %** |  |  |  | **Value** | **Percentage** | **Summed %** |
| Residence(base=Rural) |  |  |  |  |  |  |  |  |  |  |  |  |
| Urban | 0.0289 | 0.0398 | 0.7660 | 0.0304 | 32.03 | 32.03 | -0.0491 | -0.0551 | 0.7616 | -0.0420 | -42.27 | -42.27 |
| Maternal age (base=15-19 years) |  |  |  |  |  |  |  |  |  |  |  |  |
| 20-34 years | 0.0039 | 0.0061 | 0.1070 | 0.0006 | 0.68 | 0.68 | -0.0188 | -0.0351 | 0.0637 | -0.0022 | -2.25 | -2.25 |
| 35-49 years | 0.0131 | 0.0097 | 0.0085 | 0.0000 | 0.00 |  | 0.0005 | 0.0003 | -0.0234 | 0.0000 | 0.00 |  |
| Maternal education (base= No education) |  |  |  |  |  |  |  |  |  |  |  |  |
| Primary | 0.0600*** | 0.1211 | -0.3900 | -0.0472 | -49.65 | 31.56 | -0.1439 | -0.1846 | -0.3924 | 0.0724 | 72.87 | -15.29 |
| Secondary and higher | 0.0902*** | 0.1446 | 0.5344 | 0.0773 | 81.21 |  | -0.0772 | -0.2059 | 0.4256 | -0.0876 | -88.16 |  |
| Maternal occupation (base= Not working) |  |  |  |  |  |  |  |  |  |  |  |  |
| Professional work | 0.0386* | 0.0289 | 0.2378 | 0.0068 | 7.23 | 6.60 | -0.0411* | -0.0483 | 0.3072 | 0.0148 | -14.93 | -11.74 |
| Nonprofessional work | 0.0014 | 0.0017 | -0.3448 | -0.0006 | -0.63 |  | -0.0527** | -0.0290 | -0.1093 | 0.0031 | 3.19 |  |
| Partner education (base= No education) |  |  |  |  |  |  |  |  |  |  |  |  |
| Primary | 0.0286* | 0.0429 | -0.3942 | -0.0169 | -17.78 | 20.59 | 0.0815* | 0.0753 | -0.3500 | -0.0263 | -26.51 | 52.3 |
| Secondary and Higher | 0.0331* | 0.0745 | 0.4897 | 0.0365 | 38.37 |  | 0.0705 | 0.2134 | 0.3672 | 0.0783 | 78.81 |  |
| Sex of the head of household(base=Male) |  |  |  |  |  |  |  |  |  |  |  |  |
| Female | -0.0008 | -0.0006 | -0.0475 | 0.0000 | 0.00 | 0.00 | -0.0116 | -0.0170 | -0.0223 | 0.0003 | 0.38 | 0.38 |
| Household family size(base=1-3) |  |  |  |  |  |  |  |  |  |  |  |  |
| 4-6 | 0.0105 | 0.0187 | -0.0476 | -0.0008 | -0.93 | 1.15 | 0.0103 | 0.0209 | 0.0142 | 0.0002 | 0.30 | 1.55 |
| >=7 | 0.0179 | 0.0308 | 0.0642 | 0.0019 | 2.08 |  | -0.0304 | -0.0356 | -0.0350 | 0.0012 | 1.25 |  |
| Household wealth index (base=Poorest) |  |  |  |  |  |  |  |  |  |  |  |  |
| Poorer | 0.0228 | 0.0166 | -0.3342 | -0.0055 | -5.83 | 24.11 | 0.0011 | 0.0010 | -0.2848 | -0.0002 | -0.29 | 54.42 |
| Middle | 0.0282* | 0.0211 | -0.0669 | -0.0014 | -1.49 |  | 0.0228 | 0.0195 | 0.0985 | 0.0019 | 1.93 |  |
| Wealthier | 0.0312 | 0.0253 | 0.2430 | 0.0061 | 6.46 |  | 0.0768 | 0.0557 | 0.3697 | 0.0206 | 20.71 |  |
| Wealthiest | 0.0319 | 0.0318 | 0.7474 | 0.0237 | 24.97 |  | 0.0990 | 0.0611 | 0.5219 | 0.0318 | 32.07 |  |
| Read newspaper (base= No) |  |  |  |  |  |  |  |  |  |  |  |  |
| Yes | -0.0201 | -0.0125 | 0.1758 | -0.0021 | -2.30 | -2.30 | 0.0267 | 0.0369 | 0.4436 | 0.0163 | 16.17 | 16.17 |
| Listened to the radio (base= No) |  |  |  |  |  |  |  |  |  |  |  |  |
| Yes | -0.0091 | -0.0158 | 0.3128 | -0.0049 | -5.19 | -5.19 | -0.0076 | -0.0169 | 0.1846 | -0.0031 | -3.14 | -3.14 |
| Watched television (base= No) |  |  |  |  |  |  |  |  |  |  |  |  |
| Yes | 0.0158 | 0.0204 | 0.6677 | 0.0136 | 14.31 | 14.31 | 0.0627** | 0.0925 | 0.6215 | 0.0575 | 57.86 | 57.86 |
| Explained |  |  |  |  |  | 123.54 |  |  |  |  |  | 107.99 |
| Residual |  |  |  |  |  | -23.54 |  |  |  |  |  | -7.99 |
| Total |  |  |  |  |  | 100 |  |  |  |  |  | 100 |

*Note: Significance level ***p < 0.001, **p < 0.01, *p < 0.05*
