# Supplementary material for: Measuring socioeconomic inequalities in prenatal HIV test service uptake for prevention of mother to child transmission of HIV in East Africa: A decomposition analysis
Source: PLoS One. 2022 Aug 23;17(8):e0273475. doi: 10.1371/journal.pone.0273475 (PMC9398021; doi:10.1371/journal.pone.0273475)
Supplement: Attachment — Submitted filename: Response to Reviewers.docx [file pone.0273475.s003.docx]

Date: 02/06/2022

**Title:** **Measuring socioeconomic inequalities in prenatal HIV test Service uptake for prevention of mother to child transmission of HIV in East Africa: A decomposition analysis**

**Dear editor and Reviewers:** We really thank you for your valuable comments for the betterment of our manuscript. Your concerns and questions as well as suggestions are addressed in the revised manuscript (see the point-by-point response).

**Editor comment**

1. Please ensure that your manuscript meets PLOS ONE's style requirements

- ***Author response****: We have revised it*

2. Your ethics statement should only appear in the Methods section of your manuscript.

- ***Author response****: We have corrected as per your comment and incorporated in the method section.* ***See page 5 line 158-160***

3. Some minor occurrence of overlapping text with your previous publication, which needs to be addressed:

- ***Author response****: We have revised it.*

**Reviewers' comments:**

**Reviewer #1**

1. Please explain abbreviations at the first time you use them. E.g. MTCT in the abstract, PMTCT & SES in the introduction. are not explained the first time they are mentioned. Terms explained already in the introduction should then use abbreviations thereafter – I realize you still used “prevention of mother-to-child transmission of HIV” under Materials & methods.

***Author response:*** *The manuscript has been edited to reflect the reviewer concern*

2. The phrase “PMTCT of HIV” can be avoided if you define ‘PMTCT’ to include HIV, but you have not defined PMTCT.

***Author response:*** *The manuscript has been edited* *to reflect the reviewer comment.*

3. move paragraphs 3,4,5 to be the first, second & third paragraphs, respectively as these nicely introduce the main health issue and then zoom into the specific topic of the paper.

***Author response:*** *We have re-arranged and added more paragraphs in the introduction section to reflect the reviewer concern.*

4. Here I recommend listing the East African countries which were included in the 81% estimate (I recommend this because some countries like Mozambique, Zambia & Zimbabwe are sometimes grouped under Southern Africa in global statistics reports).

**Author response:** *We have listed those countries (see page 3-line number 84-88). Regarding* *countries classification, as you have said there are different geographical classification East Africa countries. In this study we selected the United Nation Statistical Division and World Health Organization classification of East Africa region as mostly health related report and studies considered this classification. We have cited the following reference to indicate list of East Africa countries in the manuscript method section (see page 5 line 132-136).*

- *United Nations Department of Economic and social affairs statistical division. Standard country or area codes for statistical use 2020 [Available from:* [*https://unstats.un.org/unsd/methodology/m49/*](https://unstats.un.org/unsd/methodology/m49/)
- United Nation. World Population Review 2020 [Available from: <https://worldpopulationreview.com/country-rankings/east-african-countries>

*Therefore, the most recent data from ten countries in WHO regions of East Africa with completed DHS datasets between 2011 and 2017 were included in this study.*

5. The fourth paragraph would start with the sentence: “Measuring socioeconomic inequalities in health service use can guide the focus of researchers and assist policymakers to prioritize specific health care needs (38)”. This way you can reduce current paragraphs 1 & 2 into a single paragraph (new paragraph 4) to be less broad but focus on defining socioeconomic inequalities and how they are measured. This paragraph would touch on one or two examples of methods for measuring these inequalities – thus an opportunity to introduce important terms such as “decomposition analysis’ which you mention in your objectives paragraph, and ‘concentration indices’ which you mention in the methods, but both not explained there. Some sentences can be easily moved from the Methods section, e.g. “CI is a popular tool to measure health and health care inequality in the field of health policy and health economics research (56).” AND “The concentration index (CI) was used to measure the extent/magnitude of socioeconomic inequalities in prenatal HIV test service utilization (54, 55).” The current first two paragraphs have some useful content but some of it is not necessary and makes the intro wander away slightly.

***Authors response:*** *Point appreciated, we have re-arranged the paragraphs and revised the introduction section more to reflect the reviewer concern.*

6. RESULTS: It might be useful to the reader in understanding why some percentages do not add up to 100%, if you indicate with a character like * in Table 1 those variable/country cells where there was missing information. E.g., Burundi ‘Urban = 8.7% and rural = 81.0%.

***Authors response:*** *To clarify how we calculated the percentage; we did not include non-user in the percentage calculation. We only calculated the percentage distribution of services users across factors. For example, the total services users’ mothers in Burundi was 89.7%, of which 8.7% of was from Urban and 81.0% was from rural. In this case, the sum of percentages is expected to be equals to the total percentage of services users in each country.*

7. DISCUSSION: Could some of the inter-country differences somehow influence the differences in results between countries? I would imagine the surveys conducted in 2011-2012 (Comoros & Mozambique) would be not comparable to those conducted in 2016 and beyond given the change in PMTCT guidelines over time. Authors may review changes in PMTCT guidelines around prenatal HIV testing across the survey years and discuss whether this could have impacted on the survey data. The authors may decide whether this is a simple discussion point or a limitation.

**Author response:** *We did adjust for countries/years in the paper while decomposing CI to socioeconomic determinants to control its effect. We assign a "countries/years” code such as country =1, 2, 3, etc. to each country. Then created a dummy variable and included in the model specification as presented in Table 2* ***(see page 18).*** *Furthermore, as noted in discussion section, survey time was indicated as one of a reason for the observed variation in socioeconomic inequalities across countries as measured in concentration index* ***(see page 19, line number 407-409).***

8. Discretionary Revisions: At the author’s choice – would using South-East and East Africa be more accurate given the geographical location of Mozambique, Zambia and Zimbabwe is accurately South-East Africa and the latter two are commonly grouped under Southern Africa as well.

***Author response:*** *As noted above,* *we* *selected the United Nation Statistical Division and World Health Organization classification of East Africa region as mostly health related report and studies considered this classification.*

**Reviewer #2:**

9. I would suggest focusing the intro on the topic and not on explaining what inequalities and inequities are. That could be a paragraph with references.

***Author response:*** *We have re-arranged and added more paragraphs in the introduction section to reflect the reviewer concern.*

10. The intro could explain why the topic is relevant and we already know about it. As the authors stated, there is already evidence of differences by socioeconomic status in HIV testing, so what is the value-added of characterizing those using the CI? How this metric could contribute to informing better policies? That is, for example, knowing that income level is a relevant contributor to testing inequalities could modify policies?

***Author response:*** *We have incorporated under the introduction section* ***(see page 4-5, line number 102-129)***

11. In terms of the data, how using a period of 7 years of surveys could affect the analysis? That is, during this period (2011-2018), there were interventions to improve testing in those countries, in particular in the countries with the earliest surveys?

***Author response:*** *As noted above, we did adjust for countries/years in the paper while decomposing CI to socioeconomic determinants to control its effect. We assign a "countries/years” code such as country =1, 2, 3, etc. to each country. Then created a dummy variable and included in the model specification as presented in Table 2. However as noted under discussion section, survey time was indicated as one of a reason for the observed variation in socioeconomic inequalities across countries as measured in concentration index* ***(see page 19, line number 407-409).***

12. How comparable is the wealth index across countries? That is, the metric is relative to households in the country, not between countries. Also, my understanding is that the WI uses a set of variables that could differ county by country as some assets are less or more relevant depending on the context.

***Author response:*** *Since the study was in East Africa, it is impossible to assume homogeneity across countries. However, to ensure consistent and comparability of our estimates, we elected to use only the DHS dataset of each country as DHS uses similar and standardized data collection tools across countries. In addition, Wealth index quantiles were created independent for each country before pooling the data together to account wealth index inter county variation. Furthermore, the creation and adjustment of population-level weight and country-specific cluster and strata was used for each country to have a nationally representative sample in this study. The use of the same methodology (in our case concentration index decomposition approach) for each East Africa countries, also allows us to compare across country, regardless of populations, mean and distribution of the dependent variable and also mean and distribution of the socioeconomic status measure variable.*

13. The WI was used as a continuous measure or the quintiles?

***Author response****: We used as quantile wealth index as we discussed in the method section* ***(see page 7, line number 193-197).***

14. There is a? mark on formula 3 and the text that follows

***Authors response****: I think, it might have happened when the online submission system automatically converts the word document into PDF format during submission. I checked, it does not exist on the word document*

15. It seems to me that the authors interpretation of the CI is not adequate. The CI is a measure of the degree of inequality, that is, how concentrated is the health indicator among individuals with higher (lower) socioeconomic level. The value of the CI is not a percentage difference between two categories (rich/poor) as it is estimated on the complete spectrum of SE level.

***Authors response:*** *I think it might be a misunderstanding****.*** *As you have said CI is a measure of the degree of inequality, that is, how concentrated is the health indicator among individuals with higher (lower) socioeconomic level. We used SE to infer the standard error, not the percentage difference between the poor and rich category.*

16. The interpretation on the decomposition also seems not adequate. The coefficients are the relation between the CI for testing and the CI for the explanatory variable, so indicates how the inequality on the explanatory variable is related to inequality in testing.

***Authors response****: We have explained on* ***page 16, line number 354-359***

17. Interpretations as a change of residence to decrease inequalities seem not informative. **Authors response**: *We have used varied approaches to interpret the decomposition analysis result. As indicated on table 2, the decomposition analysis result was presented in terms of marginal effect, elasticity, concentration index of covariates, and percentage contribution of covariates in relation to the inequalities. Specifically, for interpretation of residence the marginal effect and elasticity of residence was used* ***(see page 15, line number 331-340).***
18. The discussion could be improved by discussing the findings using the framework of the social determinants of health, rather than trying to discern how listening to the radio or watching tv are related to testing.

***Author response:*** *We have incorporated your comment in the method section.*

We appreciate the editor and reviewers for the comments and time taken to read our manuscript.

Thank you.

Feleke Hailemichael
